# Enhancing Yield, Physiological, and Quality Traits of Strawberry Cultivated Under Organic Management by Applying Different Non-Microbial Biostimulants

**DOI:** 10.3390/plants14050712

**Published:** 2025-02-26

**Authors:** Michele Ciriello, Antonio Pannico, Youssef Rouphael, Boris Basile

**Affiliations:** Department of Agricultural Sciences, University of Naples Federico II, 80138 Portici, Italy; michele.ciriello@unina.it (M.C.); antonio.pannico@unina.it (A.P.); youssef.rouphael@unina.it (Y.R.)

**Keywords:** organic agriculture, net photosynthesis, biostimulants, physiological mechanisms, *Fragaria* × *ananassa* Duch., antioxidant activity

## Abstract

Organic farming is an environmentally friendly management practice that excludes the use of synthetic inputs, but at the same time is associated with lower yields than conventional production. In an attempt to compensate for yield reduction, resulting from foregoing the use of synthetic fertilizers, we hypothesized that the use of biostimulant products could provide much-desired food security. In light of this, a greenhouse experiment was conducted to compare and evaluate the effects of the foliar application of three different non-microbial biostimulants (a seaweed extract, a plant protein hydrolysate, and a plant extract) on the yield, mineral profile, and physiological response of strawberry (*Fragaria* × *ananassa* Duch.) grown in an organic farming context. Regardless of the type of biostimulant, treated plants showed significant improvement in photosynthetic performance. Specifically, the application of plant-derived protein hydrolysate increased ACO_2_ by 34.5% compared with control. Despite this, only the application of plant-derived protein hydrolysate significantly increased fruit yield per unit area (+13.5%). The improved performance of plants treated with plant-derived protein hydrolysate was associated with an overall improvement in mineral profile (compared to control +49.4 and 33.0% in NO_3_^−^ and Mg^2+^ concentration, respectively). In contrast, application of the seaweed biostimulant increased (+17.4%) fruit antioxidant activity (DPPH) compared with control plants. These results underscore how the diverse origins of non-microbial biostimulants are responsible for specific responses in crops that can be exploited by organic growers to increase productivity.

## 1. Introduction

The massive use of synthetic agrochemicals such as pesticides and fertilizers has contributed significantly to the chemical and physical degradation of agricultural soils, water pollution, greenhouse gas emissions, and a gradual depletion of resources [1]. Due to the critical nature of these issues, the Food and Agriculture Organization of the United Nations (FAO) has expressed the need to seek alternative intensification methods based on the conservation of nonrenewable resources [2]. In response to the environmental damage caused by chemical-dependent agricultural models, modern organic farming was established. According to European Community directives, organic farmland should cover 20% of the arable land in the European Union (EU) by 2030 [3]. The growing interest in organic agricultural products is mainly attributable to the ability to produce food while drastically reducing the damage to agroecosystems and minimizing the use of external inputs [4]. In addition to greater intrinsic environmental sustainability, the transition to organic farming would promote improved food security, especially in terms of nutrition, but also improved profits for individual farmers. In more developed countries, the emergence of international and local certifications and/or labels that uniquely recognize organic products has significantly improved the welfare and profitability of small producers [5]. However, considering production volumes, which are significantly lower than what can be achieved in conventional production settings, organic farming would not fully espouse the three pillars of sustainability (economic, environmental, and social) [4].

Several meta-analyses have highlighted the production gap between organic and conventional farming [6,7,8]. The reported results show that the limited ability to control biotic pressure and adequately supply essential nutrients reduces yields by about 25%. The bioavailability of key macro-nutrients supplied through organic fertilizers most often does not coincide with actual crop demand causing an inevitable slowdown in crop growth and development rates. In light of this, it would be necessary to allocate more land area to produce the same amount of food as conventional agriculture, incentivizing deforestation processes that would drastically reduce the benefits of organic farming. For this reason, the introduction of technological tools compatible with the methodologies allowed in organic farming is necessary.

More sustainable crop management is essential in the current agricultural scenario. In light of this complicated and alarming situation, there is a need for the rapid adoption of sustainable methods that can increase crop production and quality while improving the durability and adaptability of cropping systems [9]. The use of biostimulant-acting products has been considered one of the most promising methods to increase plant resilience to abiotic environmental challenges. By definition, the efficacy of biostimulants is not attributable to the presence of macro- and/or micro-nutrients but to the activation of specific metabolic networks capable of up-regulating the primary and secondary metabolism of plants under both optimal and non-optimal conditions [10]. The current proposed classification of biostimulant products is limited to the origin of the raw materials used, not considering the biological activity exhibited. Specifically, biostimulants have been classified into five main groups: algae and plant extracts, humic substances, protein hydrolysates, microorganisms, and inorganic compounds with biostimulant action [11]. Relative to the major non-microbial biostimulants (protein hydrolysates, plant, and algal extracts), the biostimulatory action is related primarily to the presence of specific signal molecules that trigger gene regulatory mechanisms that can result in phenotypic responses that culminate in improved productivity [12]. In any case, it should be emphasized that the chemodiversity of these molecules is strongly influenced by the type of biostimulant. In organic production settings, characterized by low inputs, the targeted use of specific biostimulants could improve the availability and uptake of nutrients by plants [1]. As widely reported in the literature, improvement in the nutritional status of plants treated with biostimulants may be related to (i) improved solubility and mobility of nutrients in the rhizosphere, (ii) changes in root architecture, and (iii) enhancement of soil microbial and enzymatic activities [13,14].

Globally, strawberry cultivation has experienced an exponential surge in recent decades especially in the northern hemisphere [15]. At the beginning of the decade, global strawberry production exceeded 8 million tons with a counter-economic value of about USD 22 million [16]. The world’s leading producer is China, followed by the United States and Egypt, while at the European level the largest producer is Spain. Growing strawberries in a protected environment, through the use of greenhouses, allowed growers to deseasonalize their production while ensuring a higher market price [17]. In today’s horticultural landscape, the cultivation of strawberries (*Fragaria* × *ananassa*) requiring high inputs (for proper nutritional and phytosanitary management) has made this fruit one of the “richest” in pesticide residues. Precisely for this reason, in recent years there has been an increased interest from both the scientific community and the commercial production world in the cultivation of strawberries under organic regimes and the concomitant use of biostimulants to improve production performance. Not surprisingly, a steady conversion of conventional strawberry cultivation to organic cultivation is still taking place in Italy [17]. In addition to the well-known negative effects related to the misuse of pesticides, both on human health and the environment, the selling price of organically grown strawberries turns out to be about 60% higher than that for conventional production [18]. However, as with all other agricultural products, organic strawberry production faces abiotic and/or biotic threats that significantly limit productivity. In light of this, the objective of our research was to evaluate, in an organic context, how the application of different non-microbial biostimulants can improve the production responses of strawberries grown in protected culture.

## 2. Results and Discussion

### 2.1. Strawberry Growth and Fruit Yield Variables as a Function of Biostimulant

**Figure 1:**
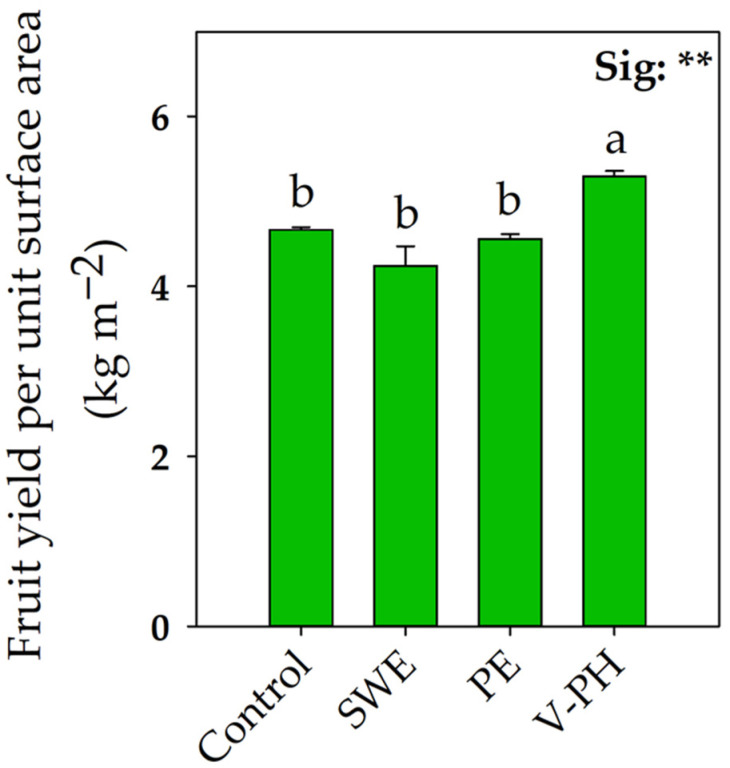
Effect of foliar applications of seaweed extract (SWE), vegetable-derived protein hydrolysate (V-PH), and plant extract (PE) on fruit yield per unit ground surface area of greenhouse-grown organic strawberry. Different letters indicate significant mean differences according to Tukey’s HSD test (*p* = 0.05). ** indicates significant effects at *p* ≤ 0.01. Data are mean values ± standard error, n = 3.

As widely reported in the literature, the interaction between different pre-harvest factors can significantly influence the development, growth, fruit yield, and quality of crops [19]. As shown in Figure 1, the application of different biostimulants influenced fruit yield per unit area of organically grown strawberries. Contrary to our hypothesis, the applications of seaweed extract (SWE) and plant extract (PE) did not significantly affect fruit yield compared with control conditions. As suggested by Nardi et al. [20], in some situations, environmental aspects such as field conditions in relation to biostimulant type may contribute to non-response to biostimulants, justifying our results. In contrast, the application of vegetable-derived protein hydrolysate (V-PH) resulted in a 13.5% increase in this crucial parameter. This result is not surprising since several studies have shown that foliar application of V-PH can enhance crop yield by selective activation of metabolic pathways involved in carbon metabolism (malate dehydrogenase, isocitrate dehydrogenase, and citrate synthase) [21,22,23]. A recent study on strawberries used metabolomic analysis to show that the application of V-PH significantly increased the content of tyrosine and phenylalanine, amino acids that actively regulate plant development and growth [2]. In an organic farming cropping system, where macro- and micro-nutrients are usually available in lower concentrations, root development and growth would play a key role in nutrient acquisition processes and fruit yield potential [24]. In these contexts, it has been shown that the application of non-microbial biostimulants such as V-PH and SWE can promote more vigorous root growth by facilitating the exploration and uptake of nutrients confined in the rhizosphere [1,25,26]. In any case, our results show a clear relationship between fruit yield improvement and biostimulant type (Figure 1).

The nonunique improvement in strawberry yield following biostimulant application could be related to deep and complex relationships between biostimulant, genotype, and environment that most often define the actual efficacy of the biostimulant [27]. In general, biostimulants due to the presence of organic target molecules promote the growth and resilience of crops to abiotic stresses. In any case, due to the wide heterogeneity of the chemical composition of non-microbial biostimulants and the complexity associated with plant growth regulation processes, it can be hypothesized that different biostimulants enact distinct mechanisms of action depending on the growth conditions [1]. The close relationship between genotype and biostimulant was highlighted by Masny et al. [28] specifically on strawberry. Namely, the foliar application of a seaweed extract resulted in significant yield improvement for the cultivar “Elkat”, while no differences were observed for the cultivar “Salut” compared to the control. As shown in Table 1, all fruit yield components (fruit number and mean fresh fruit weight) and biometric (shoot dry weight and leaf number) parameters were found to be significantly affected by biostimulant application. Unlike what was observed for fruit yield (Figure 1), the application of V-PH did not result in significant differences in number and mean fruit weight compared to the control (Table 1). Conversely for the parameters of leaf number and shoot dry weight, the application of both V-PH and PE resulted in a significant increase compared to the control (an average of 27.7 and 46.9%, respectively). Although the exogenous application of PE did not result in a significant improvement in strawberry production compared with control conditions, the biostimulant based on tropical plant extracts (PE) significantly increased the number of leaves (+29.5%) and shoot dry weight (+46.8%) (Table 1). These results once again confirm how the different origins and/or compositions of the tested biostimulants define non-unique plant responses. Specifically compared to SWE treatment, PE application reduced the number of fruits per plant (−6.9%) but significantly increased the mean weight of individual fruits (+15.6%) (Table 1). Considering that consumers tend to prefer larger strawberry fruits, producers seek useful strategies to improve fruit yield and fresh weight while minimizing sorting losses [29].

Probably in the specific case of PE, the improvement in vegetative performance may have “hindered” the flowering phase due to excessive vegetative luxuriance, as confirmed by the significant reduction in the number of fruits per plant. In contrast, V-PH-treated plants might have exploited differently the increased photosynthate production provided by improved vegetative biomass (source organs). Not surprisingly, the typical organic compounds in the V-PH tested act as signaling molecules that can up-regulate the biosynthesis of endogenous phytohormones such as gibberellins, auxins, and cytokinins [2,30]. Increased fruit production and plant biomass were similarly found in the study conducted by Ertani et al. [31] on greenhouse-grown chili peppers.

### 2.2. Strawberry Photosynthetic, Ionic and Antioxidant Response as a Function of Biostimulant

The co-presence of organic compounds, in commercial formulations of non-microbial biostimulants, can act directly and/or indirectly as signaling molecules that trigger crucial molecular and physiological processes in plants by improving production performance and mitigating the deleterious effects of abiotic stressors on crops [1]. In the present study, the application of the different biostimulants tested significantly affected all the physiological parameters except for the SPAD index and transpiration E (Table 2). Regardless of the type, the use of biostimulant products resulted in higher net CO_2_ assimilation (ACO_2_) and Fv/Fm values than the control. In contrast, the highest values of stomatal conductance (gs) and intrinsic water use efficiency (WUEi) were recorded from plants treated with SWE and V-PH and V-PH, respectively (Table 2). Compared with the control, the improvement in Fv/Fm, gs, and ACO_2_ values observed in plants treated with the seaweed-based biostimulant (SWE) could be attributable to a reduction in the inhibition of chlorophyll degradation partly driven by the presence of betaine-like compounds [32]. As suggested by Khan et al. [33], betaines, in addition to their involvement in the reduction of chlorophyll pigment degradation, are important compatible solutes actively involved in the mitigation of osmotic stresses and may represent a secondary source of nitrogen. In any case, it is important to note that improved photosynthetic performance did not result in fruit yield improvement in SWE-treated strawberry plants. The plants treated with this biostimulant probably exploited the increased availability of photosynthates to enhance antioxidant defenses (activation of secondary metabolism). In addition to this, as suggested by Rouphael et al. [34], the application of the tested biostimulants may have reduced oxidative stress at the reaction centers involved in photosynthetic processes by supporting even under nutrient-limited conditions an improved osmotic balance, more efficient stomatal movement, and consequently better diffusion of ambient CO_2_ into the chloroplasts [35]. In contrast, the fruit yield improvement, confirmed by improved photosynthetic performance, shows a more pronounced enhancement of primary metabolism triggered by V-PH application. Unsurprisingly, among all biostimulants, only V-PH significantly (+24.9%) increased WUEi compared to control (Table 2). The improved water status could be related to the presence in the commercial formulation of the already well-known bioactive peptide called “root hair promoting peptide” isolated by Matsumiya and Kubo [36]. This peptide would lead to an increase in the density and branching of the root system, improving the plant’s ability to explore a larger volume of soil and absorb water and nutrients [4].

Regarding the implications of the effect of different biostimulants on the ionic profile of strawberry leaves (Table 3), significant differences were found for NO_3_^−^, PO_4_^3−^, Ca^2+^, and Mg^2+^ content, while no significant effects were recorded among foliar applications of biostimulants for K^+^ and SO_4_^2−^ content. Regardless of biostimulant application, among the macro-elements analyzed, K^+^ was found to be the most abundant, with values ranging from 15.4 to 17.7 g/kg dw, followed by Ca^2+^ (3.5–5.6 g/kg dw), PO_4_^3−^ (2.7–3.5 g/kg dw), Mg^2+^ (2.1–2.9 g/kg dw), NO_3_^−^ (0.9–1.4 g/kg dw), and SO_4_^2−^ (0.6–0.9 g/kg dw) (Table 3).

Compared with what has been reported in the literature, the lower values of ions detected in strawberry leaves (especially K^+^) could be attributable to the non-use of synthetic fertilizers that characterize organic cultivation [37,38]. Compared with the untreated control, the significant increase in NO_3_^−^ content in V-PH-treated strawberry leaves is confirmed by the numerous studies that have shown how exogenous application of vegetable-derived protein hydrolysates can increase crop yield due to activation of metabolic pathways involved in nitrogen metabolism [21,39]. Schiavon et al. [23] and Ertani et al. [40] demonstrated how the application of V-PH can stimulate the activity of enzymes involved in nitrate assimilation processes such as aspartate aminotranferase, glutamine synthetase, glutamate synthetase, nitrite reductase, and nitrate reductase. The ability to enhance nutrient acquisition, which is often linked to the action of non-microbial biostimulants such as V-PH, may play an even more crucial role in organic farming systems where macro- and micro-nutrient availability is often limited. The higher yield values (Figure 1) recorded in strawberry plants treated with V-PH may be related not only to an improvement in nitrate assimilation but also to a higher foliar content of Mg^2+^ and Ca^2+^. The latter plays a crucial role in the adaptation to changes in nutrient status and nutrient sensing [41]. In fact, in addition to being essential for plant development and growth, Ca^2+^ directly influences numerous physiological activities and plant-environment relationships as a secondary messenger of several transduction pathways [42]. Mg^2+^ is a key macro-element for chlorophyll production, so the increased Mg^2+^ values recorded in leaves may justify the higher photosynthetic capacity and fruit yield obtained in V-PH-treated plants. Mg actively participates in photon capture and subsequent energy transfer from light-harvesting complexes (LHCs) to photosystem II (PSII) [41]. The same authors point out how Mg^2+^ is involved in photosynthate transport and thus the breakdown of carbohydrates between source and target plant organs.

**Figure 2:**
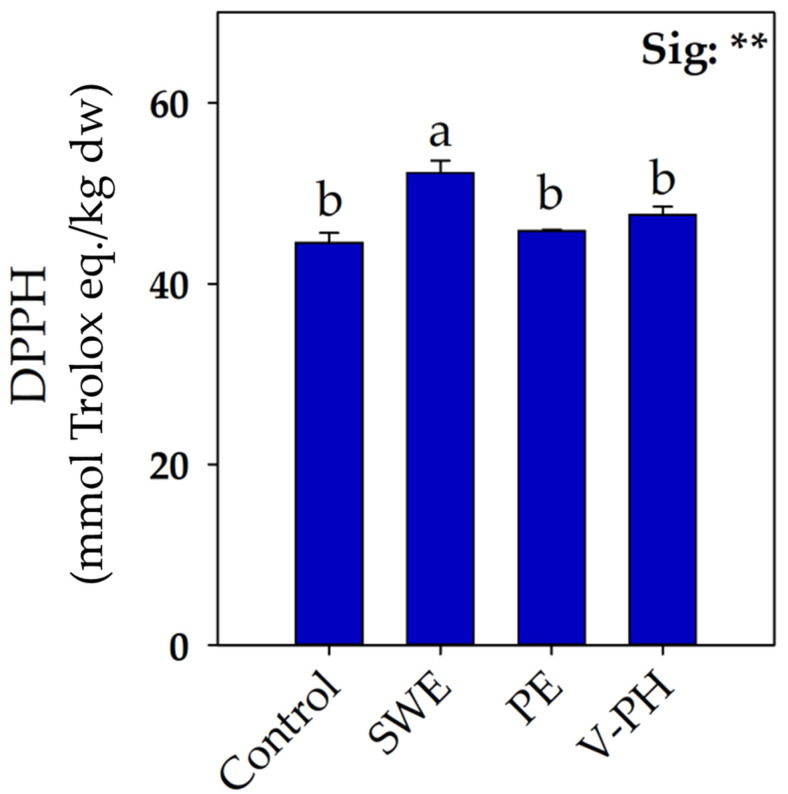
Effect of foliar applications of seaweed extract (SWE), vegetable-derived protein hydrolysate (V-PH), and plant extract (PE) on antioxidant activity (DPPH) of greenhouse-grown organic strawberry fruit. Different letters indicate significant mean differences according to Tukey’s HSD test (*p* = 0.05). ** indicates significant effects at *p* ≤ 0.01. Data are mean values ± standard error, n = 3.

Abiotic stresses such as limited nutrient availability promote biochemical processes related to photorespiration leading to H_2_O_2_ generation [43]. However, the initial overproduction of oxidative species does not cause immediate damage to the cellular mechanism since plants perceive this increase as an alarm signal that triggers adaptive responses, such as the production of specific antioxidant compounds [44]. Analysis of the radical-scavenging activity of 2,2-diphenyl-1-picrylhydrazyl (DPPH) of strawberry fruits revealed a completely different trend from that described for fruit yield. As shown in Figure 2, the highest DPPH values were recorded in strawberry fruits treated with SWE. In light of these results, it is possible to hypothesize that, differently from what was observed for V-PH and PE, the application of SWE enhanced metabolic pathways closely related to secondary metabolism.

In the specific case of SWE, the improved photosynthetic performance and the resulting increased availability of photosynthates may have been utilized by SWE-treated plants for the production of secondary metabolites that actually derived from the primary metabolic pathways [45]. As widely reported in the literature, SWEs can promote the antioxidant response of plants by reducing under stress conditions (such as essential nutrient limitation) lipid peroxidation mediated by reactive oxygen species (ROS) [1]. The higher DPPH value may be related to the different compositions of the algal biostimulant tested. Indeed, polyphenols and especially mannitol, present at high concentrations in seaweed biostimulants, are recognized as scavengers of ROS and may act as valuable antioxidants [46]. The regulation of stress tolerance triggered by foliar application of SWE might have involved the activities of antioxidants and the expression of endogenous stress-responsive genes [47]. As suggested by De Pascale et al. [4] and Ramya et al. [48], SWE application may have stimulated increased nitrogen assimilation and contextually improved nutritional status. The presence of specific bioactive compounds might also have stimulated faster plant recovery from abiotic stresses due to their role as cofactors in antioxidant activity [49]. In addition, Nair et al. [50] emphasizes the role of SWEs as osmoprotectants that can improve proline content and total soluble sugars useful in overcoming oxidative stress conditions [50]. As suggested by Drobek et al. [51], a higher antioxidant capacity would grant strawberry fruit greater protection from the deleterious effects of oxidative stress and likely improve postharvest durability.

### 2.3. Principal Component Analysis of Yield Parameters, Ionic Content, Physiological and Antioxidant Activity on Strawberry Treated with Different Non-Microbial Biostimulants

**Figure 3:**
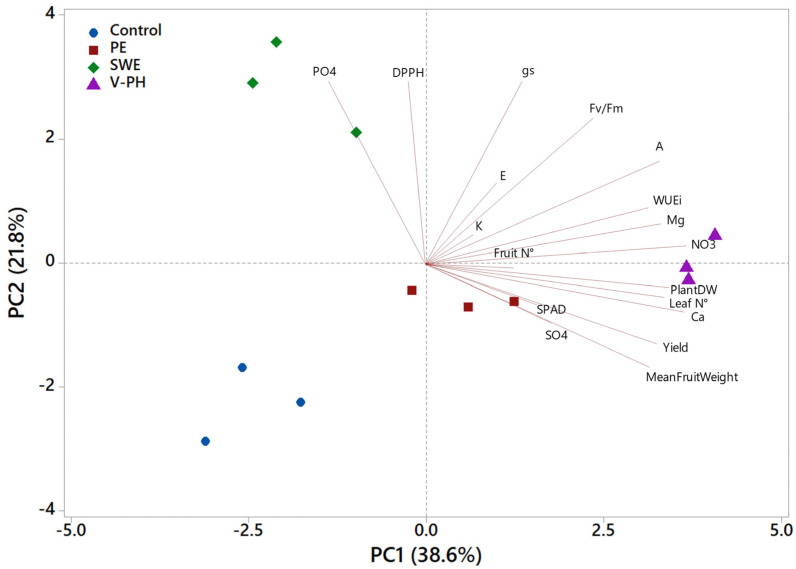
Principal component loading plot and scores of the first and second principal components (PC1 and PC2) after principal component analysis (PCA) of biometric and yield parameters, mineral content, photosynthetic performance, and antioxidant activity of organic strawberry under different foliar treatments with biostimulants (control, SWE, V-PH, and PE).

To establish a comprehensive overview of the action of the different biostimulant treatments tested on organic strawberry production, a principal component analysis (PCA) was performed on all variables reported and discussed above. The first two principal components (PCs) were associated with Eigenvalues greater than 1 and explained a total of 60.4% of the variance, with PC1 and PC2 accounting for 38.6% and 21.8%, respectively (Figure 3). PC1 was positively correlated with nitrate, calcium, and magnesium content, fruit yield per unit area, average fruit weight, plant dry weight, and major physiological parameters (ACO_2_ and WUEi). In contrast, PC2 was found to be positively correlated with the parameters of gs and E and PO_4_^3−^ content and DPPH values. The V-PH and PE treatments were located on the negative side of PC2 and on the positive side of PC1 (in the lower right quadrant of the PCA score chart), encompassing the main production and biometric parameters and SPAD, Ca, and SO_4_^2−^ values. The negative side of PC1 corresponded to the remaining treatments (control and SWE). Specifically, the upper left quadrant, which includes the SWE treatment, is characterized by higher PO_4_^3−^ content and the highest DPPH values. The present PCA facilitated an integrated view of yield and physiological variables, strengthened by the results of ion chromatography, which would allow the different actions of the different biostimulants tested to be evaluated for organic strawberry production.

## 3. Materials and Methods

### 3.1. Plant Material and Experimental Design

The experimental activity was conducted in a polyethylene tunnel greenhouse at the organic farm “Dambrosiobio” located in Succivo (Caserta, Italy) (lat. 40°98′49″ N, 14°26′41″ E, 35 m a.s.l.). On 14 October 2020, strawberry cultivar ’Sabrina’ stolons (Planasa Inc., Valtierra Navarra, Spain) were transplanted at a density of 6.5 plants/m^2^. As described by the producers, this cultivar is particularly well adapted to southern environments and is characterized by early ripening, high yield, and marked rusticity. Soil chemical and physical analyses were reported in Table 4. For the determination of soil chemical characteristics, soil samples were oven-dried at 40 °C. Regarding nitrogen (N) content, organic N content was determined by the Kjeldahl method [52]. Determination of available phosphorus was evaluated by the Olsen method [53], while for potassium, determination was performed by spectrophotometry at 650 nm, as described in detail by Sunderman [54]. Organic matter was determined according to the methodology proposed by Walkley and Black [55].

Irrigation, fertilization, and pest and disease management were carried out following organic farming practices [56]. In accordance with the organic regime, green manure with *Vicia faba* (L.) was planted, without using preplant fertilization. Before transplanting, the soil was tilled and arranged in mulched raised beds. A drip system with one dripper per plant (1.2 L/h) was set up below the mulch for irrigation and fertigation management. Irrigation volumes and frequency were managed according to the estimated evapotranspiration and phenological stage of the crop. Fertigation involved the use of organo-mineral fertilizers allowed in organic farming applied every 10–15 days at a dose of 20 kg/ha. The dose of organic nitrogen was reduced after flowering, preferring potassic and calcium and micro-nutrient organic fertilizers. During the crop cycle, all early flowers were removed from strawberry plants to prolong fruit harvest. To prevent disease, the relative humidity in the tunnels was monitored and controlled by managing the openings in order to provide adequate ventilation. Pest and disease control has been carried out on a weekly basis by combining preventive measures, natural predators, organic sprays, and biological controls according to organic farming practices [56].

**Figure 4:**
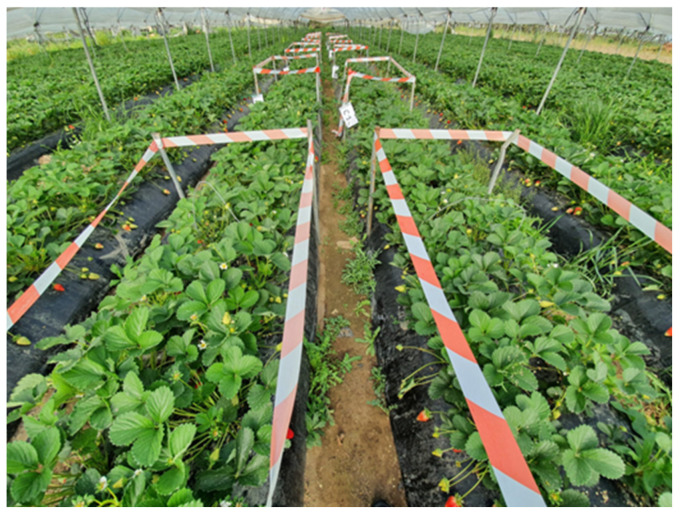
Representative picture of the experimental trial.

The experimental trial involved a comparison between the application of three non-microbial biostimulants of different origins and an untreated control. The biostimulants used were a vegetable protein hydrolysate [hereafter V-PH, Trainer, Hello Nature, Verona, Italy], a plant extract [hereafter PE, Auxym, Hello Nature], and a seaweed extract [hereafter SWE, ED&F Man, Bologna, Italy]. The commercial biostimulant V-PH was obtained by enzymatic hydrolysis and contains 75% free amino acids and peptides, 22% carbohydrates, and 3% mineral nutrients. The results for the aminogram (Ala, Arg, Asp, Cys, Glu, Gly, His, Ile, Leu, Lys, Met, Phe, Pro, Ser, Thr, Trp, Tyr, and Val) of the product along with the phenolic compound content and elemental composition have been reported in detail by Paul et al. [57]. Further analysis did not record the presence of detectable phytohormones. PE-based biostimulant was produced by water extraction with fermentation of tropical plant biomass. This product contains mainly phytohormones (auxins and cytokinins), amino acids, peptides, vitamins (C and E, niacin, pyridoxine, riboflavin, and thiamine), and essential micro-nutrients (B, Cu, Fe, Mn, and Zn). According to the information reported by the manufacturer, the seaweed-based biostimulant product (SWE) has N and organic carbon contents of 1% and 10%, respectively, while the auxin, cytokinin, and levorotatory amino acid contents were 10 mg/L, 0.028 mg/L, and 11%, respectively. From February 12 (121 days after transplantation, DAT) each biostimulant was applied every 10 days by foliar application at the recommended doses: 3.0, 2.0, and 2.0 mL/L for V-PH, PE, and SWE respectively. The doses used in the present experiment are in accordance with what has been reported in the literature in similar experiments [58,59]. All biostimulants plus the untreated control were replicated three times for a total of 12 experimental units and arranged in a randomized design. Each experimental unit consisted of 10 plants for a total of 30 plants per treatment (Figure 4).

### 3.2. Fruit Yield, Physiological Parameters, Fruit Quality, and Leaf Ion Content Analyses

Starting from March 5 (142 DAT), 17 fruit harvests were carried out. Total fruit yield per unit ground surface area (kg/m^2^) was determined at the end of the experiment (236 DAT). The number of fruits and leaves per plant was determined individually for each plant. At the end of the experiment, ten plants per replication were placed in a ventilated stove (M40-VF, Artiglass, Padova, Italy) for the determination of dry plant biomass (g plant^−1^). Physiological measurements were carried out on fully expanded young leaves of six plants per replicate at 180 DAT. Net CO_2_ assimilation (ACO_2_; μmol CO_2_/m^2^/s), transpiration (E; mmol H_2_O/m^2^/s), and stomatal conductance (gs; mol H_2_O/m^2^/s) were measured using a LI-6400 portable gas exchange analyzer (LI-COR Biosciences, Lincoln, NE, USA). The intrinsic water use efficiency (WUEi) was subsequently calculated as the ratio of ACO_2_ to E. On the same leaves, the values of Fv/Fm and SPAD index were determined using a handheld fluorometer Fv/Fm Meter (Plant Stress Kit, Opti-Sciences, Hudson, NH, USA) and a handheld chlorophyll meter SPAD-502 (Minolta Corp. Ltd., Osaka, Japan), respectively. On previously dried plant material, the content of cations (K^+^, Ca^2+^, and Mg^2+^) and anions (NO_3_^−^, PO_4_^3−^ and SO_2_^4−^) was determined by ion chromatography (Thermo Scientific™ Dionex™, Sunnyvale, CA, USA). Briefly, 0.25 g of dry, finely ground plant tissue was weighed and then mixed with 50 mL of ultrapure water. The obtained samples were frozen, thawed, and centrifuged (6000 rpm, 10 min; Remi Elektrotechnik Ltd., Mumbai, Maharashtra, India). The supernatant obtained after injection was filtered and processed by ion chromatography coupled to an electrical conductivity detector. As described in detail by Formisano, et al. [60], ion concentrations were quantified by comparing peak areas of the samples with reference standards. The radical-scavenging activity of 2,2-diphenyl-1-picrylhydrazyl (DPPH) was determined according to the protocol described by Brand-Williams et al. [61]. A 1 mL aliquot of DPPH solution was added to 200 mL of extract, mixed, and incubated for 10 min at room temperature. The absorbance was recorded at 517 nm with a UV-VIS spectrophotometer (Shimadzu, Kyoto, Japan). For all instrumental analyses, each treatment was analyzed in triplicate

### 3.3. Statical Analysis

All data (Supplementary Material) were analyzed using IBM SPSS Statistics software (version 26.0, SPSS Inc., Chicago, IL, USA). All mean effects were subjected to one-way ANOVA analysis. Statistical significance between treatments was determined by Tukey’s HSD test. Principal component analysis (PCA) of biometric and yield parameters, mineral content, photosynthetic performance, and antioxidant activity was performed to discriminate the effects of the different biostimulants under study, using Minitab 16.2.1 statistical software.

## 4. Conclusions

The lower yields achievable in biological environments inevitably clash with the need to increase food production for an ever-increasing population. This paradox has driven research toward identifying sustainable methods and strategies that can minimize this critical issue. The results of our research highlight how the choice of the proper non-microbial biostimulant type can play a positive role in organic strawberry production contexts. Although the use of all three non-microbial biostimulants tested (PE, V-PH, and SWE) improved the photosynthetic performance of strawberry (*Fragaria* × *ananassa* Duch.), only the foliar application of V-PH ensured a significant increase in fruit yield probably due to an improvement in plant nutritional status (specifically nitrate, magnesium, and calcium). In order to further optimize the use of biostimulants under unfavorable conditions (such as organic cultivation), further research is needed to deepen the understanding of the different molecular and biochemical mechanisms put in place by different biostimulants and how they interact with other pre-harvest factors (choice of genotype, growing conditions, and growing environment).

## Figures and Tables

**Table 1:** Effect of foliar applications of seaweed extract (SWE), vegetable-derived protein hydrolysate (V-PH), and plant extract (PE) on fruit number per plant, mean fruit fresh weight, leaf number per plant, and shoot dry weight per plant of greenhouse-grown organic strawberry.

Treatment	Fruit Number (N°/Plant)	Fruit Fresh Weight (g/Fruit)	Leaf Number (N°/Plant)	Shoot Dry Weight (g/Plant)
Control	35.46 ± 0.23 ab	20.23 ± 0.21 ab	55.40 ± 1.24 b	42.09 ± 3.31 b
SWE	34.56 ± 0.21 b	18.86 ± 0.85 b	56.00 ± 2.71 b	42.21 ± 1.19 b
PE	32.16 ± 0.53 c	21.80 ± 0.27 a	71.73 ± 1.95 a	61.80 ± 1.01 a
V-PH	36.90 ± 0.20 a	22.07 ± 0.35 a	69.73 ± 0.40 a	61.88 ± 1.46 a
Significance	***	**	***	***

Different letters indicate significant mean differences according to Tukey’s HSD test (*p* = 0.05). *** and ** indicate significant effects at *p* ≤ 0.001 and *p* ≤ 0.01. Data are mean values ± standard error, n = 3.

**Table 2:** Effect of foliar applications of algae extract (SWE), vegetable-derived protein hydrolysate (V-PH) and plant extract (PE) on SPAD index, net CO_2_ assimilation (ACO_2_), transpiration (E), stomatal conductance (gs), intrinsic water use efficiency (WUEi), and Fv/Fm of greenhouse-grown organic strawberry.

Treatment	SPAD Index	ACO_2_ (µmol CO_2_/m^2^/s)	E (mol H_2_O/m^2^/s)	gs (mol H_2_O/m^2^/s)	WUEi (µmol CO_2_/mol H_2_O)	Fv/Fm
Control	45.60 ± 0.23	12.05 ± 0.53 b	2.59 ± 0.18	0.15 ± 0.00 b	4.66 ± 0.18 b	0.80 ± 0.00 b
SWE	44.37 ± 0.81	14.64 ± 0.42 a	2.83 ± 0.14	0.20 ± 0.00 a	5.18 ± 0.10 ab	0.81 ± 0.00 a
PE	44.26 ± 0.77	15.22 ± 0.40 a	2.77 ± 0.09	0.17 ± 0.00 b	5.48 ± 0.09 ab	0.81 ± 0.00 a
V-PH	46.40 ± 0.64	16.24 ± 0.11 a	2.80 ± 0.16	0.19 ± 0.00 a	5.82 ± 0.30 a	0.81 ± 0.00 a
Significance	n.s.	***	n.s.	***	*	***

Different letters indicate significant mean differences according to Tukey’s HSD test (*p* = 0.05). n.s., *, and *** are non-significant or significant at *p* ≤ 0.05, and 0.001, respectively. Data are mean values ± standard error, n = 3.

**Table 3:** Effect of foliar applications of seaweed extract (SWE), vegetable-derived protein hydrolysate (V-PH), and plant extract (PE) on the mineral profile of greenhouse-grown organic strawberry.

Treatment	NO_3_^−^	SO_4_^2−^	K^+^	Ca^2+^	Mg^2+^	PO_4_^3−^
	(g/kg dw)					
Control	0.91 ± 0.08 b	0.73 ± 0.04	16.99 ± 0.27	3.84 ± 0.25 b	2.15 ± 0.03 b	2.72 ± 0.18 b
SWE	0.97 ± 0.04 b	0.64 ± 0.05	17.35 ± 0.81	3.48 ± 0.30 b	2.38 ± 0.18 ab	3.56 ± 0.12 a
PE	1.05 ± 0.05 ab	0.63 ± 0.09	15.39 ± 0.63	4.08 ± 0.22 b	2.20 ± 0.09 b	2.92 ± 0.10 b
V-PH	1.36 ± 0.10 a	0.60 ± 0.07	17.73 ± 0.60	5.62 ± 0.32 a	2.86 ± 0.02 a	2.80 ± 0.11 b
Significance	*	n.s	n.s.	**	**	**

Different letters indicate significant mean differences according to Tukey’s HSD test (*p* = 0.05). n.s., *, and ** are non-significant or significant at *p* ≤ 0.05 and 0.01, respectively. Data are mean values ± standard error, n = 3.

**Table 4:** Physical and chemical properties of soil.

Properties	Unit	Results
Sand	%	48.4
Silt	%	43
Clay	%	8.6
pH		7.4
Electrical conductivity	dS/m	0.2
Organic Matter	%	2.4
Total Nitrogen	g/kg	1.7
C/N		8.3
P_2_O_5_ (ppm)	mg/kg	162
K_2_O (ppm)	mg/kg	2439

## Data Availability

The raw data supporting the conclusions of this manuscript are reported in the Supplementary Material (Table S1).

## Supplementary Materials

The following supporting information can be downloaded at: https://www.mdpi.com/article/10.3390/plants14050712/s1, Table S1: All experimental data subjected to the analysis of variance (ANOVA).

## References

[1] Sani M.N.H., Yong J.W. (2021). Harnessing synergistic biostimulatory processes: A plausible approach for enhanced crop growth and resilience in organic farming. Biology.

[2] Cardarelli M., El Chami A., Rouphael Y., Ciriello M., Bonini P., Erice G., Cirino V., Basile B., Corrado G., Choi S. (2024). Plant biostimulants as natural alternatives to synthetic auxins in strawberry production: Physiological and metabolic insights. Front. Plant Sci..

[3] Wrzaszcz W. (2023). Tendencies and perspectives of organic farming development in the EU–the significance of European Green Deal Strategy. Eur. J. Sustain. Dev..

[4] De Pascale S., Rouphael Y., Colla G. (2017). Plant biostimulants: Innovative tool for enhancing plant nutrition in organic farming. Eur. J. Hortic. Sci.

[5] Ume C. (2023). The role of improved market access for small-scale organic farming transition: Implications for food security. J. Clean. Prod..

[6] de la Cruz V.Y.V., Cheng W., Tawaraya K. (2023). Yield gap between organic and conventional farming systems across climate types and sub-types: A meta-analysis. Agric. Syst..

[7] Lesur-Dumoulin C., Malézieux E., Ben-Ari T., Langlais C., Makowski D. (2017). Lower average yields but similar yield variability in organic versus conventional horticulture. A meta-analysis. Agron. Sustain. Dev..

[8] Ponisio L.C., M’Gonigle L.K., Mace K.C., Palomino J., De Valpine P., Kremen C. (2015). Diversification practices reduce organic to conventional yield gap. Proc. R. Soc. B Biol. Sci..

[9] Zulfiqar F., Moosa A., Ali H.M., Bermejo N.F., Munné-Bosch S. (2024). Biostimulants: A sufficiently effective tool for sustainable agriculture in the era of climate change?. Plant Physiol. Biochem..

[10] Saavedra T., Gama F., Correia P.J., Da Silva J.P., Miguel M.G., de Varennes A., Pestana M. (2020). A novel plant extract as a biostimulant to recover strawberry plants from iron chlorosis. J. Plant Nutr..

[11] Franzoni G., Cocetta G., Prinsi B., Ferrante A., Espen L. (2022). Biostimulants on crops: Their impact under abiotic stress conditions. Horticulturae.

[12] Li J., Lardon R., Mangelinckx S., Geelen D. (2024). Practical guide toward discovery of biomolecules with biostimulant activity. J. Exp. Bot..

[13] Sible C.N., Seebauer J.R., Below F.E. (2021). Plant biostimulants: A categorical review, their implications for row crop production, and relation to soil health indicators. Agronomy.

[14] Parađiković N., Teklić T., Zeljković S., Lisjak M., Špoljarević M. (2019). Biostimulants research in some horticultural plant species—A review. Food Energy Secur..

[15] Shakya R., Capilla E., Torres-Pagán N., Muñoz M., Boscaiu M., Lupuţ I., Vicente O., Verdeguer M. (2023). Effect of two biostimulants, based on Ascophyllum nodosum extracts, on strawberry performance under mild drought stress. Agriculture.

[16] (2017). Faostat. http://www.fao.org/faostat/en/#data.

[17] Righini H., Roberti R., Baraldi E. (2018). Use of algae in strawberry management. J. Appl. Phycol..

[18] Soltaniband V., Brégard A., Gaudreau L., Dorais M. (2022). Biostimulants promote plant development, crop productivity, and fruit quality of protected strawberries. Agronomy.

[19] Kouam I.D., Moungang S., Koulagna H.I., Ntsoli G.P., Titti R.W., Yaouba A. (2024). Influence of organic and mineral fertilizers and a foliar biostimulant on the yield and nutritional quality of strawberries (*Fragaria* × *ananassa* Duch.) under field conditions. Biochem. Syst. Ecol..

[20] Nardi S., Pizzeghello D., Schiavon M., Ertani A. (2016). Plant biostimulants: Physiological responses induced by protein hydrolyzed-based products and humic substances in plant metabolism. Sci. Agric..

[21] Pasković I., Popović L., Pongrac P., Polić Pasković M., Kos T., Jovanov P., Franić M. (2024). Protein Hydrolysates—Production, Effects on Plant Metabolism, and Use in Agriculture. Horticulturae.

[22] Malécange M., Sergheraert R., Teulat B., Mounier E., Lothier J., Sakr S. (2023). Biostimulant properties of protein hydrolysates: Recent advances and future challenges. Int. J. Mol. Sci..

[23] Schiavon M., Ertani A., Nardi S. (2008). Effects of an alfalfa protein hydrolysate on the gene expression and activity of enzymes of the tricarboxylic acid (TCA) cycle and nitrogen metabolism in *Zea mays* L.. J. Agric. Food Chem..

[24] Möller K. (2018). Soil fertility status and nutrient input–output flows of specialised organic cropping systems: A review. Nutr. Cycl. Agroecosystems.

[25] Rajesaheb K.S., Subramanian S., Boominathan P., Thenmozhi S., Gnanachitra M. (2024). Bio-stimulant in improving crop yield and soil health. Commun. Soil Sci. Plant Anal..

[26] Garg S., Nain P., Kumar A., Joshi S., Punetha H., Sharma P.K., Siddiqui S., Alshaharni M.O., Algopishi U.B., Mittal A. (2024). Next generation plant biostimulants & genome sequencing strategies for sustainable agriculture development. Front. Microbiol..

[27] Li J., Van Gerrewey T., Geelen D. (2022). A meta-analysis of biostimulant yield effectiveness in field trials. Front. Plant Sci..

[28] Masny A., Basak A., Żurawicz E. (2004). Effects of foliar applications of Kelpak SL and Goëmar BM 86® preparations on yield and fruit quality in two strawberry cultivars. J. Fruit Ornam. Plant Res..

[29] Wise K., Selby-Pham J. (2024). Strawberry field trial in Australia demonstrates improvements to fruit yield and quality control conformity, from application of two biostimulant complexes. N. Z. J. Crop Hortic. Sci..

[30] Mrid R.B., Benmrid B., Hafsa J., Boukcim H., Sobeh M., Yasri A. (2021). Secondary metabolites as biostimulant and bioprotectant agents: A review. Sci. Total Environ..

[31] Ertani A., Pizzeghello D., Francioso O., Sambo P., Sanchez-Cortes S., Nardi S. (2014). Capsicum chinensis L. growth and nutraceutical properties are enhanced by biostimulants in a long-term period: Chemical and metabolomic approaches. Front. Plant Sci..

[32] Ali O., Ramsubhag A., Jayaraman J. (2021). Biostimulant properties of seaweed extracts in plants: Implications towards sustainable crop production. Plants.

[33] Khan W., Rayirath U.P., Subramanian S., Jithesh M.N., Rayorath P., Hodges D.M., Critchley A.T., Craigie J.S., Norrie J., Prithiviraj B. (2009). Seaweed extracts as biostimulants of plant growth and development. J. Plant Growth Regul..

[34] Rouphael Y., Carillo P., Cristofano F., Cardarelli M., Colla G. (2021). Effects of vegetal-versus animal-derived protein hydrolysate on sweet basil morpho-physiological and metabolic traits. Sci. Hortic..

[35] Tränkner M., Tavakol E., Jákli B. (2018). Functioning of potassium and magnesium in photosynthesis, photosynthate translocation and photoprotection. Physiol. Plant..

[36] Matsumiya Y., Kubo M. (2011). Soybean peptide: Novel plant growth promoting peptide from soybean. Soybean and Nutrition.

[37] Tabatabaei S., Yusefi M., Hajiloo J. (2008). Effects of shading and NO_3_: NH_4_ ratio on the yield, quality and N metabolism in strawberry. Sci. Hortic..

[38] Li H., Li T., Fu G., Katulanda P. (2013). Induced leaf intercellular CO_2_, photosynthesis, potassium and nitrate retention and strawberry early fruit formation under macronutrient limitation. Photosynth. Res..

[39] Sun W., Shahrajabian M.H., Kuang Y., Wang N. (2024). Amino acids biostimulants and protein hydrolysates in agricultural sciences. Plants.

[40] Ertani A., Cavani L., Pizzeghello D., Brandellero E., Altissimo A., Ciavatta C., Nardi S. (2009). Biostimulant activity of two protein hydrolyzates in the growth and nitrogen metabolism of maize seedlings. J. Plant Nutr. Soil Sci..

[41] de Bang T.C., Husted S., Laursen K.H., Persson D.P., Schjoerring J.K. (2021). The molecular–physiological functions of mineral macronutrients and their consequences for deficiency symptoms in plants. New Phytol..

[42] Jing T., Li J., He Y., Shankar A., Saxena A., Tiwari A., Maturi K.C., Solanki M.K., Singh V., Eissa M.A. (2024). Role of calcium nutrition in plant Physiology: Advances in research and insights into acidic soil conditions-A comprehensive review. Plant Physiol. Biochem..

[43] Das K., Roychoudhury A. (2014). Reactive oxygen species (ROS) and response of antioxidants as ROS-scavengers during environmental stress in plants. Front. Environ. Sci..

[44] Hasanuzzaman M., Parvin K., Bardhan K., Nahar K., Anee T.I., Masud A.A.C., Fotopoulos V. (2021). Biostimulants for the regulation of reactive oxygen species metabolism in plants under abiotic stress. Cells.

[45] Hussein R.A., El-Anssary A.A. (2019). Plants secondary metabolites: The key drivers of the pharmacological actions of medicinal plants. Herb. Med..

[46] Iwamoto K., Shiraiwa Y. (2005). Salt-regulated mannitol metabolism in algae. Mar. Biotechnol..

[47] Calvo P., Nelson L., Kloepper J.W. (2014). Agricultural uses of plant biostimulants. Plant Soil.

[48] Ramya S.S., Vijayanand N., Rathinavel S. (2015). Foliar application of liquid biofertilizer of brown alga Stoechospermum marginatum on growth, biochemical and yield of Solanum melongena. Int. J. Recycl. Org. Waste Agric..

[49] Van Oosten M.J., Pepe O., De Pascale S., Silletti S., Maggio A. (2017). The role of biostimulants and bioeffectors as alleviators of abiotic stress in crop plants. Chem. Biol. Technol. Agric..

[50] Nair P., Kandasamy S., Zhang J., Ji X., Kirby C., Benkel B., Hodges M.D., Critchley A.T., Hiltz D., Prithiviraj B. (2012). Transcriptional and metabolomic analysis of Ascophyllum nodosum mediated freezing tolerance in Arabidopsis thaliana. BMC Genom..

[51] Drobek M., Cybulska J., Frąc M., Pieczywek P., Pertile G., Chibrikov V., Nosalewicz A., Feledyn-Szewczyk B., Sas-Paszt L., Zdunek A. (2024). Microbial biostimulants affect the development of pathogenic microorganisms and the quality of fresh strawberries (*Fragaria ananassa* Duch.). Sci. Hortic..

[52] Bremner J. (1965). Total nitrogen. Methods of Soil Analysis: Part 2 Chemical and Microbiological Properties.

[53] Olsen S.R. (1954). Estimation of Available Phosphorus in Soils by Extraction with Sodium Bicarbonate.

[54] Sunderman F.W., Sunderman F.W. (1958). Studies in Serum Electrolytes XXII. A Rapid, Reliable Method for Serum Potassium Using Tetraphenylboron. Am. J. Clin. Pathol..

[55] Walkley A., Black I.A. (1934). An examination of the Degtjareff method for determining soil organic matter, and a proposed modification of the chromic acid titration method. Soil Sci..

[56] (2020). Sicily Region. Regional Integrated Production Regulations: Technical Standards for Integrated Crop Protection and Pest Control. https://www.regione.sicilia.it/sites/default/files/2023-07/2%20Norme%20tecniche%20Difesa%202023%20del%20DPI%20Sicilia_0.pdf.

[57] Paul K., Sorrentino M., Lucini L., Rouphael Y., Cardarelli M., Bonini P., Miras Moreno M.B., Reynaud H., Canaguier R., Trtílek M. (2019). A combined phenotypic and metabolomic approach for elucidating the biostimulant action of a plant-derived protein hydrolysate on tomato grown under limited water availability. Front. Plant Sci..

[58] Ciriello M., Campana E., Colla G., Rouphael Y. (2024). An Appraisal of Nonmicrobial Biostimulants’ Impact on the Productivity and Mineral Content of Wild Rocket (*Diplotaxis tenuifolia* (L.) DC.) Cultivated under Organic Conditions. Plants.

[59] Colla G., Cardarelli M., Bonini P., Rouphael Y. (2017). Foliar applications of protein hydrolysate, plant and seaweed extracts increase yield but differentially modulate fruit quality of greenhouse tomato. HortScience.

[60] Formisano L., Ciriello M., El-Nakhel C., De Pascale S., Rouphael Y. (2021). Dataset on the effects of anti-insect nets of different porosity on mineral and organic acids profile of *Cucurbita pepo* L. fruits and leaves. Data.

[61] Brand-Williams W., Cuvelier M.-E., Berset C. (1995). Use of a free radical method to evaluate antioxidant activity. LWT-Food Sci. Technol..

